# Spatial, Temporal, and Demographic Patterns in the Prevalence of Hemorrhagic Septicemia in 41 Countries in 2005–2019: A Systematic Analysis with Special Focus on the Potential Development of a New-Generation Vaccine

**DOI:** 10.3390/vaccines10020315

**Published:** 2022-02-17

**Authors:** Reyad Almoheer, Mohd Effendy Abd Wahid, Hidayatul Aini Zakaria, Mohd Anuar Bin Jonet, Muhanna Mohammed Al-shaibani, Adel Al-Gheethi, Siti Nor Khadijah Addis

**Affiliations:** 1 Faculty of Science and Marine Environment, Universiti Malaysia Terengganu, Kuala Nerus, Bandar Kuala Terengganu 21030, Terengganu, Malaysia; p4208@pps.umt.edu.my; 2 Faculty of Fisheries and Food Sciences, Universiti Malaysia Terengganu, Kuala Nerus, Bandar Kuala Terengganu 21030, Terengganu, Malaysia; effendy@umt.edu.my; 3 Faculty of Ocean Engineering Technology and Informatics, Universiti Malaysia Terengganu, Kuala Nerus, Bandar Kuala Terengganu 21030, Terengganu, Malaysia; hidayatul@umt.edu.my; 4 Malaysia Genome and Vaccine Institute, National Institutes of Biotechnology Malaysia, Jalan Bangi, Kajang 43000, Selangor, Malaysia; jonetanuar@gmail.com; 5 Micropollutant Research Centre (MPRC), Faculty of Civil Engineering & Built Environment, Universiti Tun Hussein Onn Malaysia, Parit Raja, Batu Pahat 86400, Johor, Malaysia; muhanna@uthm.edu.my

**Keywords:** prevalence, hemorrhagic septicemia, *Pasteurella multocida*, virus-like particles (VLPs), vaccine

## Abstract

Hemorrhagic septicemia (HS) caused by *Pasteurella multocida* B:2 and E:2 is among the fatal bacterial diseases in cattle and buffaloes that are economically valuable in Asian and African countries. The current work aims to study the prevalence of HS among buffaloes, cattle, sheep, and goats in 41 countries in 2005–2019. The data analysis revealed that 74.4% of the total infection rate in the world was distributed among cattle, followed by buffaloes (13.1%). The mortality of HS among cattle and buffaloes increased in 2017–2019 compared to the period between 2014 and 2016. The best measure to control the disease is through vaccination programs. Current commercial vaccines, including live-attenuated vaccines and inactivated vaccines, have some shortcomings and undesirable effects. Virus-like particles (VLPs) have more potential as a vaccine platform due to their unique properties to enhance immune response and the ability to use them as a platform for foreign antigens against infectious diseases. VLPs-based vaccines are among the new-generation subunit vaccine approaches that have been licensed for the human and veterinary fields. However, most studies are still in the late stages of vaccine evaluation.

## 1. Prevalence of Hemorrhagic Septicemia

*Pasteurella multocida*, is a Gram-negative (size of 0.2–0.4 × 0.6–2.5 μm), non-motile, nonspore-forming, capsulated short rod or coccobacillus, and facultative anaerobic bacterium [1,2]. It has been classified as an opportunistic pathogen that causes various diseases such as hemorrhagic septicemia (HS) of buffaloes and cattle, poultry fowl cholera, purulent rhinitis of rabbits, atrophic rhinitis of swine, and enzootic pneumonia of sheep and goats. In addition, the disease also occurs in American bison, camels, yaks, elephants, deer, horses, hippopotamuses, minks, cats, monkeys, lions, tortoises, snow leopards and elks [1,3,4,5]. The disease is transmitted by ingestion of contaminated grass and water or by inhaling the bacterium [6]. At the same time, the infection is observed in humans with a history of domestic animal contact, mostly by bite or scratch from a cat or a dog that can cause lymphangitis and cellulitis [7,8,9,10].

Serologically, *P. multocida* strains are recognized in five serogroups (A, B, D, E, and F) depending on capsule antigens, which can be determined using a passive (indirect) haemagglutination test [11,12]. In addition, the bacterium strains have 16 somatic antigen serotypes based on the structure of lipopolysaccharide antigens, which can be detected by the agar gel immunodiffusion precipitation test, and also eight genotypes of lipopolysaccharides (LPS) (L1–L8) as determined by multiplex PCR [11,13]. HS is caused by type B: 2 and E: 2 strains, atrophic rhinitis is caused by type D strain, while fowl cholera is caused by type A: 1 and A: 3 strains [14].

HS is a highly lethal, acute septicemic disease caused by one of two specific serotypes of *P. multocida* designated as B: 2 (Asian serotype) and E: 2 (African serotype). The disease mainly occurs in water buffaloes and cattle, but occasionally in pigs and feral ruminants [1,15]. Buffaloes show a shorter course of the disease and are more susceptible to HS than cattle with a mortality rate reaching 100% in untreated cases at an early stage, which is often impossible in current field conditions [1,16]. Furthermore, HS has been detected in most regions in the world, especially Asia and Africa, where the disease is endemic [15]. In contrast, the estimated global population of buffalo is 204.3 million, with 97.09% found in Asia, followed by Africa (1.7%), America (0.97%), and Europe (0.22%), whereas the estimated global population of cattle is 1.5 billion, with 34.9% distributed in America, followed by 31.1% in Asia, 23.8% in Africa, and 7.8% in Europe [17]. The disease is commonly associated with rainy weather, high humidity, poor husbandry conditions, and immunosuppression [1,6,18]. The relationship between infection and immunity among different ages of the animals has not been reported in detail. However, the disease is uncommon in calves under six months, which might be because the animals have sufficient maternal immunity at these ages [1]. In comparison, in endemic areas, most deaths are related to aged calves and young adults, whereas massive epizootics occur in non-endemic areas and are responsible for 30% of estimated deaths of cattle worldwide [19,20].

HS is one of the most economically important bacterial diseases of animals, especially in tropical regions [20] such as South Asia and South-east Asia, the Middle-East, Central, North-East, and South Africa [6,21]. The estimated economic losses to the livestock industry is USD 792 million per year [22]. In the same context, economic losses from bovine death in Pakistan and India are estimated to be approximately USD 1.15 billion [23]. According to the World Organization for Animal Health (OIE), HS is classified within list B as a highly transmissible disease [24].

The most common symptoms of HS are typically febrility, lethargy, hypersalivation, nasal discharge, lacrimation, diarrhea, difficulty breathing, swelling in the submandibular region, and septic shock with widespread hemorrhage. In most cases, the infected animals die within two to three days [16,20]. Antimicrobial therapy (tetracyclines, sulfonamides, kanamycin, penicillin, streptomycin, trimethoprim, gentamicin, and chloramphenicol) is only effective during early infection. Mortality among animals with acute clinical symptoms reached 100% due to the high resistance to antibiotics among bacterial strains. Therefore, the vaccines against HS represent the best way to avoid severe bacterial diseases [20,25,26].

Despite the availability of various traditional vaccines on the market, the search for appropriate extensively protective and safe HS vaccines with long-lasting immunity continues [27]. The present paper aims to study the prevalence of HS among buffaloes, cattle, sheep, and goats in 41 countries in the period of 2005 and 2019. In addition, this paper highlights the current development of HS vaccines and future prospects of subunit vaccines to be used as a vaccine platform against HS, which will assist researchers in designing targeted virus-like particle (VLP)-based vaccines, particularly in animal vaccination.

**Figure 1:**
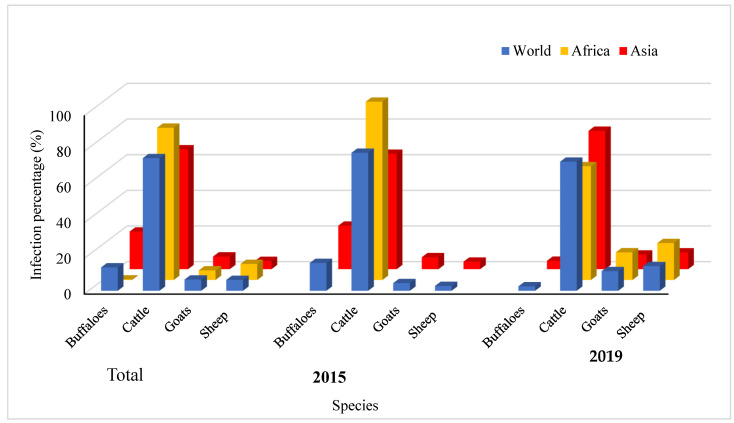
Spatial distribution of HS among buffaloes, cattle, goats and sheep in the period between 2005 and 2019 and total percentage.

The data used in the current systematic analysis were extracted from the World Organization for Animal Health (OIE-WAHIS) for the prevalence of HS among buffaloes, cattle, sheep, and goats in 41 countries in 2005–2019. The total recorded cases were 105,692,984, of which 48,454,663 (45.8%) were classified as susceptible. Meanwhile, the total death cases were 99,550, representing 0.1% of the total cases and costing USD 692,092.90 annually. The data analysis revealed that 74.4% of the total infection rate in the world was distributed among cattle, followed by buffaloes. In Africa, 85.4% and 9% of the total infection rate were recorded among cattle and sheep, respectively. In Asia, the infection rate among cattle was 67.2% and it was 21.1% among buffaloes, whereas in America and Europe, the infection rate was 100% among cattle (Figure 1).

The temporal distribution of HS among buffaloes, cattle, goats, and sheep between 2005 and 2019 is depicted in Figure 1. The infection rate among cattle worldwide dropped from 77.4% in 2005 to 72.4% in 2019, while the infection rate among buffaloes declined from 15.6% in 2005 to 2.8% in 2019. In Africa, the infection rate among cattle decreased from 100% in 2005 to 63.8% in 2019, whereas the infection rate among sheep and goats increased to 20.7% and 15.5%, respectively, in 2019. In Asia, the infection rate among cattle, sheep, and goats increased from 64.7%, 4.2%, and 6.7% in 2005 to 77.6%, 9.4% and 8.2% in 2019, respectively, while the infection rate among buffaloes decreased from 24.4% in 2005 to 4.7% in 2019.

**Figure 2:**
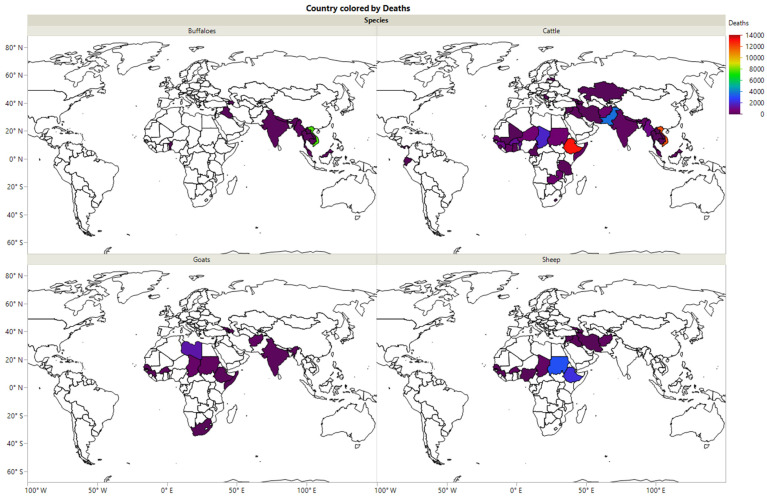
The morbidity of HS among buffaloes, cattle, goats and sheep in different countries during the period 2005–2019.

The distribution of HS among buffaloes, cattle, goats, and sheep in different countries is presented in Figure 2. It was observed that HS among buffaloes is most common in Iraq, Azerbaijan, India, Nepal, Myanmar, Thailand, Vietnam, Malaysia, and Togo. In comparison, the morbidity of HS among cattle has a wide distribution in different countries, including Iraq, Azerbaijan, Syria, Afghanistan, Pakistan, Kazakhstan, Iran, India, Nepal, Myanmar, Thailand, Vietnam, Malaysia, Latvia, Somalia, Sudan, Tanzania, Zambia, Chad, Niger, Mali, Senegal, Guinea, Ecuador, and Panama. In addition, the morbidity of HS among goats has been reported in India, Myanmar, Afghanistan, Azerbaijan, Georgia, Somalia, Ethiopia, Sudan, Libya, Chad, Burkina Faso, Senegal, Guinea, and South Africa. In contrast, the morbidity of HS among sheep has been recorded in Iran, Afghanistan, Syria, Azerbaijan, Georgia, Ethiopia, Sudan, Chad, Nigeria, Burkina Faso, and Senegal.

**Figure 3:**
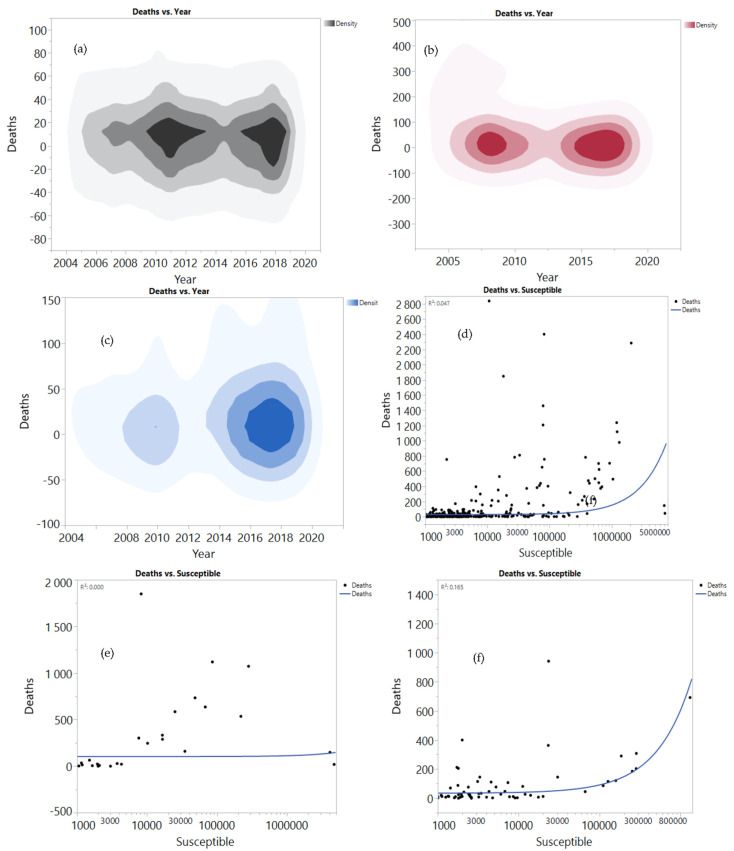

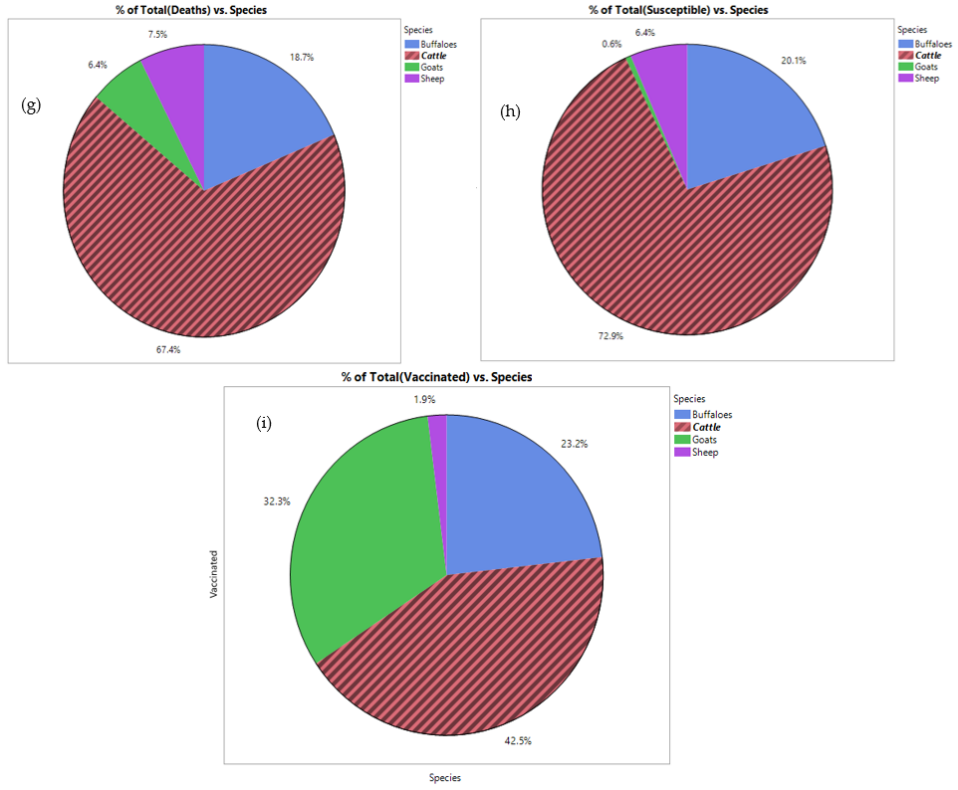
Correlation between mortality, susceptible and total morbidity among buffaloes (**a**,**d**,**g**), cattle (**b**,**e**,**h**), goat and sheep (**c**,**f**,**i**) and the total morbidity in the period between 2005 and 2019.

The data analysis for the mortality of HS among buffaloes, cattle, goats, and sheep is presented in Figure 3. Mortality among cattle increased in 2017–2019 compared to the period between 2014 and 2016 (Figure 3a). Note that, in the future, mortality is expected to be more prevalent. In comparison, the mortality rates among buffaloes increased in the last three years higher than the recorded mortality rates from 2005–2010 (Figure 3b). Mortality among sheep and goats increased in 2016 and beyond (Figure 3c). The regression analysis between mortality and the susceptible cases among buffaloes revealed that mortality increased with increasing susceptible cases (4.7%) (Figure 3d), and mortality among susceptible cattle was low (Figure 3e). On the contrary, susceptible sheep and goats recorded high mortality (R2 = 16.5%) (Figure 3f). Among the total mortality, 67.4% was recorded among cattle, followed by buffaloes with 18.7% (Figure 3g). This finding might be related to the high susceptibility between cattle (72.9%) and buffaloes (20.1%) (Figure 3h). Sheep and goats showed lower mortality (13.9%), perhaps due to lower susceptibility (7%), and a high percentage of vaccination among sheep and goats (34.2%) (Figure 3i). These results were presented for 41 counties using JMP software based on data analysis over a 14-year period. The correlation and relation between mortality, susceptibility, and total morbidity among the different species were determined.

The results provide more understanding for the direction of the HS among buffaloes, cattle, goats and sheep to highlight the need for developing an effective vaccine to minimize mortality, susceptibility, and total morbidity. Furthermore, the efficiency of the vac-cine in the reduction of HS has been reported in Europe, where the infection rate has been reduced more significantly compared to that reported in Asia and Africa. 

## 2. Virulence Factors and Associated Genes in *Pasteurella multocida* B:2 and E:2

Different important factors of virulence have been identified in *P. multocida* strains that cause HS [28]. The most significant factors include outer-membrane proteins (OMPs), capsules, lipopolysaccharides (LPS), endotoxins, and fimbriae. Additionally, genes associated with virulence are considered to be a critical factor in HS [29,30]. All *P. multocida* serotype B strains have virulence-related gene coding for type 4 fimbriae (ptfA), outer membrane proteins (OmpH, Oma87), transferrin-binding protein (tbpA), filamentous hemagglutinin (pfhA), hemoglobin-binding protein (hgbA), superoxide dismutase (sodC) and neuraminidases (nanH) [7,30,31]. Type 4 fimbriae (pili), the subunit PtfA that conserves 21-amino acids has the role of adhering bacterial cells to the mucosal epithelium surfaces of the host cell [32,33,34]. OMPs have powerful roles in virulence antigens; therefore, many OMPs have been used in the development of vaccines and as protection in experiments against bacterial pathogens [35]. OmpH is one of the OMPs that serve as antigenic surface protein and is detected in all bovine isolates; furthermore, OmpH has the potential as a vaccine candidate [31,36,37,38].

Although LPS has the main role in HS pathogenesis, it is very sensitive to the degree of killing when hosted by antimicrobial peptides [39]. Furthermore, LPS is a protective antigen because it stimulates host immune responses and produces bactericidal antibodies [20]. LPS also plays an essential role as an agent in determining 16 somatic antigens of *P. multocida* [13,40]. LPS intravenous inoculation extracted from serotype B: 2 strains can reproduce clinical symptoms of HS in buffaloes [41].

According to the classification of *P. multocida* by serological methods, there are five capsule groups of A, B, D, E, and F [12]. Generally speaking, strains with acapsular variants are less virulent than strains with capsule variants because the existence of the capsule is a significant factor that allows *P. multocida* strains to evade innate host immune defenses [4,29,42]. Although scholars have explained the significant role of the capsule as virulence determinants in the pathogenesis of *P. multocida*, acapsular mutant strains of *P. multocida* serotype B extremely attenuated from the HS infection in mice [43]. The *cexA* gene is considered a part of a 15-gene capsule (*cap*) biosynthesis locus, and has been proved to be a virulence determinant for the *P. multocida* strain M1404 (serotype B:2) in mice [43]. In addition, extracellular enzymes of *P. multocida* such as hyaluronidase, effectively contribute to increasing bacterial virulence [44]. There are many extracellular enzyme genes, with significant virulence potential against HS [20].

## 3. Vaccination against HS

Various vaccines are available on the shelf to control and prevent HS disease. These vaccines are more effective in endemic areas, and when the vaccines are used two to three months before a high-risk season [6,45]. However, despite the availability of various types of vaccines, the outbreak of HS still occurs due to improper vaccination, inadequate vaccination coverage, and low effective vaccine usage [22,45,46]. As a result, scientists try to improve vaccines to eliminate the weaknesses of current vaccines. This section discusses four types of HS vaccines, including inactivated vaccines, live vaccines, subunit vaccines, and nucleic acid vaccines. In addition, commercial vaccines incorporate adjuvants, such as alum-precipitated vaccines, aluminum hydroxide gel vaccines, oil adjuvant vaccines, and broth bacterins to enhance host immune responses [1,47].

**Figure 4:**
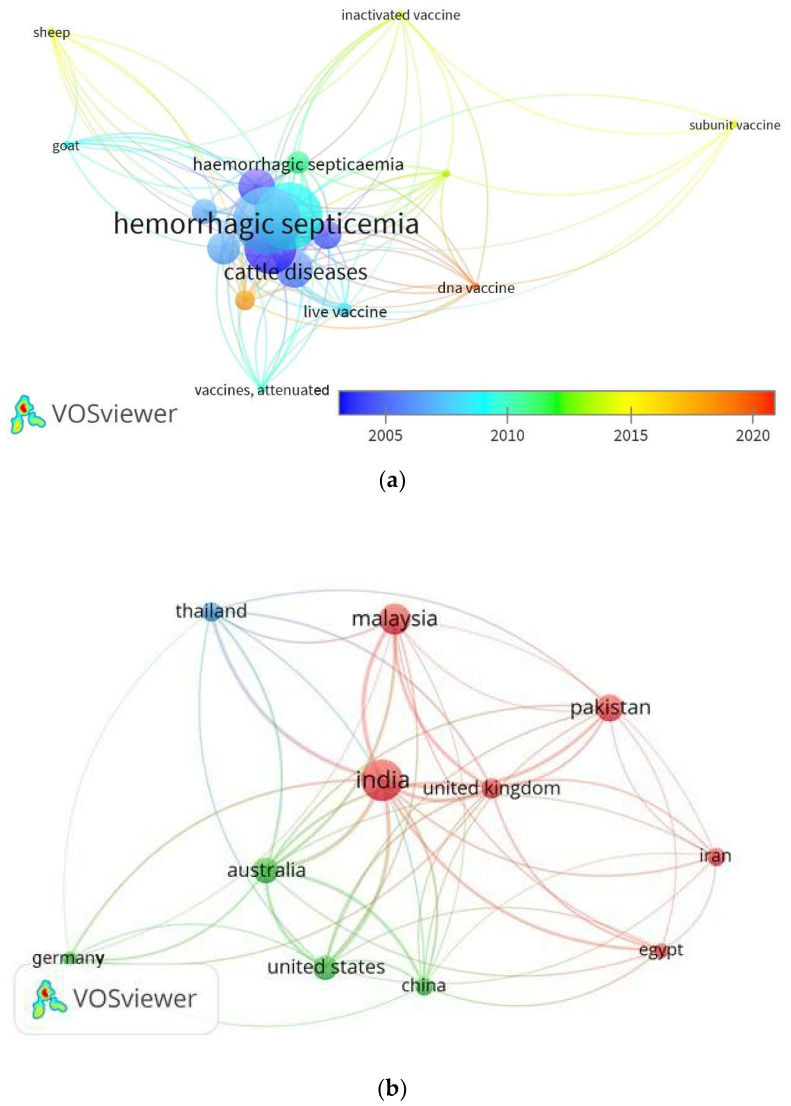
(**a**) The visualization map shows the trends in vaccine development against HS in the period between 2005 and 2020. (**b**) Visualization map shows the HS trends among the countries.

Figure 4a,b present the data analysis for vaccine development trends designed using VOSviewer software (version 1.6.15). The analysis is based on the Scopus database for studies conducted on the vaccine development between 2005 and 2021 using specific keywords of “*Pasteurella multocida*”, AND “hemorrhagic septicemia” OR “haemorrhagic septicaemia” AND “vaccine”. From the results, subunit and DNA vaccines have received great attention among scientists in the last few years (Figure 4a). Furthermore, important studies were published in India, Malaysia, and Pakistan (Figure 4b).

### 3.1. Live-Attenuated Vaccines

A live-attenuated vaccine might mimic natural infection in its early stages of development, and it is assumed that this vaccine might confer stronger and longer protective immunity [48]. According to De Alwis (1999) [1], there are different aspects of an ideal live vaccine, including having all or most of the protective antigens in the strains, easy to grow in culture media, allows the virulence strain or being avirulent for cattle and buffaloes, is stable in its virulence property, has no reversion to virulence, and the has the ability to multiply sufficiently in vivo after vaccination to produce a full complement of the main immunogens for stimulating sufficient immune responses.

*P. multocida* B: 2 was mutagenized to produce a streptomycin-dependent mutant that was highly immunogenic in mice and rabbits, as well as the protected cattle and buffaloes against HS in field trials [49]. A temperature-sensitive mutant of *P. multocida* type B proved to be stable in growth culture and was used as a live vaccination to protect calves [50]. Intranasal or intraperitoneal vaccination with the *aroA* mutant strain, which is a derivative of two *P. multocida* B: 2 strains, conferred protective immunity in mouse and calf models against HS [48,51]. On the other hand, the intranasally vaccinated calves with the *aroA* mutant strain were not protected [52]. The vaccine, which contained a live *gdhA* derivative from *P. multocida* B: 2, successfully elicited systemic immunity in exposed and in-contact buffaloes, acting as an effective live vaccination to protect both exposed and in-contact buffaloes against challenge with the virulent parent strain [25]. The freeze-drying method proved the stability of a live-attenuated vaccine *gdhA* derivative *P. multocida* B:2 as a vaccine [53]. Moreover, many studies in calves appeared to provide significant immune response and protection against HS [54,55]. A live-attenuated vaccine is better at producing prolonged cellular immunity than inactivated vaccines [56,57]. However, they may cause undesirable side effects, such as injection site abscesses and vaccine-induced diseases [21,57]. Currently, no country uses a live-attenuated vaccine to control endemic HS cases except Myanmar [20,58].

### 3.2. Inactivated Vaccines

Among the major inactivated (killed) vaccines are bacterins, alum-precipitated vaccines, aluminum hydroxide gel vaccines, oil adjuvant vaccines and multiple emulsion vaccines [59]. Bacterin is the simplest form of HS vaccine prepared from killed *P. multocida* using either physical (heat, drying, UV irradiation) or chemical agents (phenol, lysol, formalin, sodium azide) [1,21,60]. The first killed vaccine for HS was developed in 1907. However, antibody responses to the bacterin vaccine were poor, as immunity was provided for only six months and associated with a certain level of shock to animals [1,20]. Formalized or agar-wash heat-killed vaccines were enhanced to prevent protein shock caused by killed broth culture vaccine organisms, and immunity was provided for up to four months [21].

The use of alum-precipitated vaccine against HS is widespread in Asia and Africa. This vaccine contains bacterin with added potash alum to reach the final concentration of 1% [1]. The efficacy of this vaccine gives protection against HS and provides protective immunity for four to six months, but it is associated with shock reactions [1,21,61]. Among the 156 buffaloes vaccinated with the alum-precipitated vaccine and tested for the presence of anti-HS antibodies, a higher percentage of buffaloes (67.94%) exhibited a protective level of antibodies even three months after vaccination [62].

The common characteristics between the aluminum hydroxide gel vaccine and the alum-precipitated vaccine have been reported. The blending of aerated cultures with aluminum hydroxide gel led to a final concentration of 0.3% [1]. Furthermore, the conferring immunity as a result of the vaccine did not last more than 90 days, even with the use of levamisole and vitamin E as immunomodulators [21]. Aluminum hydroxide gel vaccines combined with saponin have also been used in attempts to increase immune protection [63]. This vaccine is the main type of vaccine used in Thailand and Laos [21] and can produce immunity for up to six months [1].

The first development of an oil adjuvant vaccine (OAV) against HS was reported in the 1950s [64]. This vaccine is made of water in oil emulsion, where the aqueous phase comprises a dense broth bacterial cell culture, and the oil phase consists of mineral oil (e.g., Ondina 17 and Marco l52). During vaccine preparation, equal or almost equal volumes of an aqueous phase and an oil phase need to be used. The stability of the vaccine is ensured using an emulsifying agent, such as mannite mono-oleate (Arlacel A) and lanoline [1]. This vaccine is the most effective vaccine as it confers immunity for up to one year. However, the limitation lies in the high viscosity of the vaccine solution, which makes it difficult to inject, and leads to swelling and abscesses at the injection site [38,65]. An improved OAV against HS was found to provide cross-protection against *P. multocida* E: 2,5 and B: 2,5 strains but not against B: 3,4 strains [66]. The OAV vaccine with adjuvant Montanide ISA-70 might provide promising protection in vaccinated calves [67]. In addition, the formulation of this vaccine with saponin has been shown to have induced a stronger cellular and humoral immunity against HS in calves and mice [68].

Multiple emulsion (ME) vaccines were prepared by re-emulsifying OAV with 2% Tween 80, which eased the administration and became more effective in studies related to protection. However, the immunity duration was reduced for the multi-emulsion HS vaccine [6,21]. The ME vaccine demonstrated low viscosity, ease of handling, and no side effects at the inoculation site [59]. Oil adjuvant and multiple emulsion were determined as the more effective adjuvants during the evaluation of different adjuvants in HS vaccines, including aluminum hydroxide, oil adjuvant, and multiple emulsion [20,21].

### 3.3. Nucleic Acid Vaccines

The DNA vaccine is a plasmid containing a pathogen gene that can be expressed in either mammalian cells or a gene encoding a mammalian protein (non-infectious diseases) [69,70]. This vaccine cannot reproduce, infect, or cause disease [71]. Only two DNA vaccines have been approved as veterinary vaccines: West Nile-Innovator^®^ DNA of Fort Dodge, which is licensed by the United States Department of Agriculture (USDA) for horses, and APEX-IHN^®^ of Novartis, which is licensed by the Canadian Food Inspection Agency (CFIA) as prevention for infectious hematopoietic necrosis in salmon farms [72]. In India, the outer membrane protein DNA vaccines were tested in 2011 as potential vaccines against HS of buffaloes and cattle [73]. In 2020, a DNA vaccine, pVAX1-ABA392, was produced with a high titer of anti-HS antibodies and proved to be a potential vaccine candidate against HS [74].

### 3.4. Subunit Vaccines

Subunit vaccines are composed of peptides, proteins, or polysaccharides that carry protective epitopes of the pathogen and elicit a protective immune response in the recipient [62,75]. In terms of safety, these vaccines are safer than the traditional vaccine profile because they lack replication ability in the host [70,76]. Subunit vaccines are generated as recombinant proteins using various protein expression systems, such as *E. coli*, yeast, insect and mammalian cells, and then purified and injected into a host to evoke immunity [70,77]. Subunit vaccines often require adjuvants for improving the desired immune responses [78,79]. Therefore, many studies have focused on using recombinant protein with suitable adjuvants that could potentially serve as an effective subunit vaccine against HS [80,81,82,83]. The identification of virulence and immunity genes from *P. multocida* is the important factor considered to produce recombinant vaccines [84].

There are many genes with potential as subunit vaccine candidates, which have been localized and targeted due to their involvement in virulence and immunity in *P. multocida* [84,85]. For instance, PtfA [34], capsule [43], Oma87 [30], porin (OmpH) [38], PlpE [8], HgbA [86], adhesin (OmpA) and TbpA [87,88] have been considered as candidate antigens for recombinant vaccine development to achieve a positive result for next-generation vaccines.

The current frontier areas of research have potential involvement in new technologies related to the improvement of effective new age HS vaccines to control HS [20]. A subunit vaccine using the OMPs of *P. multocida* B: 2 represents a potent candidate vaccine [89,90,91,92]. Furthermore, the expression of the *ptfA* gene of the fimbrial protein *P. multocida* serotype B: 2 was found to provide positive protection and improved immunization to HS [93,94]. The gene (*tbpA*) encoding the transferrin-binding protein from *P. multocida* serogroup B: 2 has high antigenic characteristics. It has a similar sequence to the identical gene from *P. multocida* serogroups A: 1 and D: 1 [88]. Furthermore, the virulence genes *tbpA* and *pfhA* were found to be closely associated with serotype B that caused HS [95].

Recombinant clones of ABA 392 and CSI57 J were obtained from *P. multocida* B: 2 which have been shown to provide immunity response to mice against HS [96,97]. Subsequently, the same group studied the ABA392/pET30a clone and it was tested in rats; the outcome highlighted the potential of the expressed protein as a suitable vaccine candidate against HS [98]. Recombinant proteins used as subunit vaccines are safe for immunocompromised animals as they do not cause disease [1,21] and trigger strong immunity [85]. Several potential subunit vaccine candidates against HS have been reported (Table 1). However, no commercial recombinant HS subunit vaccine has been approved until now.

### 3.5. Virus-Like Particle Vaccines

Virus-like particles (VLPs) consist of viral capsid proteins that self-assemble into the actual conformation of the respective native virus. However, VLPs do not have the capability of self-replication in cells due to the lack of the viral genome producing safer vaccine candidates even without any adjuvant and yet exhibiting increased immunogenicity [104,105,106,107].

VLPs are appealing vaccine platforms and combine various advantages, such as favorable size (10–200 nm in diameter), safety, repetitive surface geometry, ease of production, and stimulation of humoral and cellular immunity, making them better than a recombinant protein vaccine [72,108,109,110]. VLP vaccines might be used as a platform for foreign antigen epitopes against infectious diseases, which can be achieved through the incorporation of antigenic epitopes into VLPs by genetic fusion (chimeric VLPs) or through the conjugation of antigens to VLPs. The most common technique for showing heterologous epitopes on VLPs is via genetic engineering of target sequences into viral structural proteins to generate chimeric particles [72,111]. Since the mid-1980s, VLP chimeras have been investigated as potential vaccine candidates [112].

VLPs are produced in various expression systems, with more than 174 different host systems that can be utilized for the production of VLPs, including bacteria (28%), yeast (20%), mammalian (15%), plant (9%), and insect cells (28%) [113]. The choice of an appropriate expression system is critical and can have an important impact on vaccine safety, stability, and efficacy [105]. The use of optimized cultural conditions helps to produce huge quantities of VLPs more efficiently and rapidly. After successful production in the host expression system, VLPs are self-assembled into the final shape, which usually looks like the original symmetry of the virus [114]. Although VLPs have structural and intrinsic features that allow them to induce immunological responses without the use of adjuvants, the use of licensed adjuvants with VLP vaccines can improve vaccine immunogenicity and stimulate the activation of a specific type of immune response [112,115,116].

In animal vaccines, VLP technology is deemed to be one of the most attractive approaches due to its intrinsic immunogenic properties and high safety profiles [117,118]. Several VLP vaccines are licensed for commercialization in the human field, including vaccines against human papillomavirus (HPV), such as Cervarix^®^, Gardasil ^®^, and Gardasil9^®^; hepatitis E virus (HEV) (Hecolin); and hepatitis B virus (HBV) (Recombivax^®^ and Engerix^®^) [119,120]. However, there is only one VLP-based vaccine in the veterinary field, which was licensed for commercialization in 2009 against porcine circovirus type 2 (PCV2), Porcilis PCV^®^ (Intervet), while other vaccines are still in clinical trials [118,119,120]. To the best of our knowledge, this is the only report that has demonstrated feasibility in the production of murine polyomavirus-like particles containing the *P. multocida* fimbriae protein (VLP-fimbriae), and further research is required to demonstrate the potential of the VLP-fimbriae protein as a vaccine candidate against HS [121].

## 4. Conclusions

*Pasteurella multocida* B: 2 and E: 2 are the causative agent of HS that occurs mainly in water buffaloes and cattle. It has been found in almost all parts of the world as a costly animal disease that causes billions of dollars in losses annually. The data analysis in the present work revealed that 74.4% of morbidity was reported among cattle, while 13.1% was recorded among buffaloes. The HS mortality among these animals increased in 2017–2019 compared to the period between 2014 and 2016. Vaccination against HS remains the most effective way to prevent the disease, and the antibiotics are inefficient. These vaccines include live vaccines, inactivated vaccines, subunit vaccines, and nucleic acid vaccines. Furthermore, not all of these vaccines are available on the market. More than a decade after the production of the first HS vaccine, huge efforts have been made to develop an appropriate and cost-effective vaccine for HS. Nevertheless, to date, most HS vaccines used as commercial vaccines are either inactivated vaccines, such as alum-precipitated vaccine, aluminum hydroxide gel vaccine, and oil adjuvant vaccine, or live vaccines, such as broth bacterins. These commercial vaccines have several drawbacks, such as short-term immunization, reversion to virulence, insufficient cross-protection, and induced local inflammation at the site of injection. Thus, multidisciplinary cooperation, including microbiology, immunology, molecular biology, genetics, proteomics, and even bioinformatics, should be advocated in developing state-of-the-art animal vaccines. There is a greater focus in current research areas involving new-generation vaccines, particularly on the development of new and more effective HS vaccines, which can lead to the implementation of an effective HS control plan. In addition, new-generation vaccines, including recombinant protein, DNA, and VLP-based vaccines, are fulfilling this requirement, making them particularly attractive for use as animal vaccines. It is hoped that the use of new-generation vaccines against HS may soon become a reality. Recombinant proteins, such as OmpH and PtfA, have been shown to confer significant potency associated with the protection and enhancement of immunities against HS. DNA vaccine pVAX1-ABA392 and outer membrane protein produced a high titer of antibody against HS and have the potential to be vaccine candidates. VLPs are more effective as vaccine candidates due to their interesting properties, such as favorable size, safe, repetitive surface geometry, ease of production, and stimulation of both humoral and cellular immunity, indicating their potency in compression, which is better than the recombinant protein vaccine. These characteristics have attracted scientists in the past 35 years to utilize VLPs as unique tools in vaccine development strategies in the veterinary field. VLPs have been widely used in the vaccine field and there is a clear trend toward the establishment of VLPs as a powerful tool for vaccine development. VLP-based vaccines have already been licensed for human and animal diseases. Five human vaccines are already in the market for HBV and HPV and one in the veterinary field (PCV2). Many more vaccine candidates are currently in the late stages of evaluation. Utilizing VLPs in vaccine development is a rapidly growing field that combines different biomedical disciplines, including immunology, virology, microbiology, and vaccinology.

## Figures and Tables

**Table 1:** The potential recombinant subunit candidate vaccine against HS.

Recombinant Clone	Gene Size (bp)	Expressed Protein Size (kDa)	Animal Model Study for Immunogenicity	References
ptfA	435	18	rabbits	[34]
rOmpH	980	37	mice	[38]
pET32/LICfimbrial	450	33	goat	[93]
rOmpH	1002	33.7	mice	[99]
CSI57J (ABA392)	921	26	mice	[97]
pPtfA	414	31	pigs, sheep and goats	[94]
rOmpH	942	34	mice	[100]
rOmp87	2304	102	mice	[80]
pQE 30-omp87	2300	80	N/A	[101]
rVacJ	699	44	mice	[81]
rTbpA	2244	103	mice	[82]
rOmp16	411	32	mice	[83]
rOmpH	960	37	calves	[102]
ABA392-pET30a	804	32	rat	[98]
rOmpW	519	37	mice	[103]
rOmpH	960	37	buffaloes	[27]

## Data Availability

Data can be accessed and extracted using OIE-WAHIS (https://wahis.oie.int/#/home, accessed on 4 September 2021).
